# Hearing Loss and Cognitive Function in Early Old Age: Comparing Subjective and Objective Hearing Measures

**DOI:** 10.1159/000527930

**Published:** 2022-12-14

**Authors:** Maria Hoff, Johan Skoog, Timothy Hadarsson Bodin, Tomas Tengstrand, Ulf Rosenhall, Ingmar Skoog, André Sadeghi

**Affiliations:** ^a^Unit of Audiology, Department of Health and Rehabilitation, Institute of Neuroscience and Physiology, Sahlgrenska Academy, University of Gothenburg, Gothenburg, Sweden; ^b^Hearing Organization, Habilitation and Health, Region Västra Götaland, Gotaland, Sweden; ^c^Neuropsychiatric Epidemiology Unit, Department of Psychiatry and Neurochemistry, Institute of Neuroscience and Physiology, Sahlgrenska Academy, University of Gothenburg, Mölndal, Gothenburg, Sweden; ^d^EPINEP Research Group, Centre for Ageing and Health (AgeCap), University of Gothenburg, Gothenburg, Sweden; ^e^Department of Psychology, University of Gothenburg, Gothenburg, Sweden

**Keywords:** Cognitive function, Cognitive domains, Age-related hearing loss, Tinnitus, Hearing aids

## Abstract

**Introduction:**

Population-based research has consistently shown that people with hearing loss are at greater risk of cognitive impairment. We aimed to explore the cross-sectional association of both subjective and objective hearing measures with global and domain-specific cognitive function. We also examined the influence of hearing aid use on the relationship.

**Methods:**

A population-based sample (*n* = 1,105, 52% women) of 70-year-olds that were representative of the inhabitants of the city of Gothenburg, Sweden completed a detailed cognitive examination, pure-tone audiometry, and a questionnaire regarding perceived hearing problems. A subsample (*n* = 247, 52% women) also completed a test of speech-recognition-in-noise (SPRIN). Multiple linear regression analyses were conducted to explore the association of hearing with cognitive function, adjusting for sex, education, cardiovascular factors, and tinnitus.

**Results:**

Global cognitive function was independently associated with the better ear pure-tone average across 0.5–4 kHz (PTA4, β = −0.13, 95% CI, −0.18, −0.07), the better ear SPRIN score (β = 0.30, 95% CI, 0.19, 0.40), but not with the self-reported hearing measure (β = −0.02, 95% CI, −0.07, 0.03). Both verbally loaded and nonverbally loaded tasks, testing a variety of cognitive domains, contributed to the association. Hearing aid users had better global cognitive function than nonusers with equivalent hearing ability. The difference was only significant in the mild hearing loss category.

**Discussion:**

In a population-based sample of 70-year-old persons without dementia, poorer hearing was associated with poorer global and domain-specific cognitive function, but only when hearing function was measured objectively and not when self-reported. The speech-in-noise measure showed the strongest association. This highlights the importance of including standardized hearing tests and controlling for hearing status in epidemiological geriatric research. More research is needed on the role that hearing aid use plays in relation to age-related cognitive declines.

## Introduction

Hearing loss and dementia are two prevalent health conditions in advancing age that have serious negative effects on the lives of affected individuals as well as their families. Both conditions have been demonstrated to affect numerous domains of healthy aging, including social engagement, mental well-being, and daily functioning [1]. In addition, health outcomes have been found to be poorer when sensory and cognitive impairment coexist [2]. According to the World Alzheimer Report 2015, the number of people across the world living with dementia amount to approximately 50 million [3], and the equivalent figure for disabling hearing loss is as much as 500 million [4]. These figures are expected to rise dramatically in the future owing to the worldwide increase in life expectancy; consequently, both age-related hearing loss and dementia are recognized as major public health challenges. The role of cognition in hearing and auditory processing is well described in the literature. Cognitive functions, such as working memory capacity, processing speed, attention, and executive function, are important when it comes to speech perception, particularly in challenging acoustic environments [5, 6]. Furthermore, research has consistently demonstrated that hearing impairment − both of peripheral and central origin − increases the risk for mild cognitive impairment and dementia [7]. The concept of a link existing between hearing impairment and cognitive decline was suggested already in the early work by Uhlmann et al. [8] and has gained ground considerably in recent years due to a growing body of evidence from large cohort studies [9, 10, 11, 12, 13, 14, 15, 16]. Moreover, a recent report from the Lancet Commission on the prevention of dementia [17] lists hearing loss as the greatest potentially modifiable risk factor that, if managed, may delay the onset of dementia. Although no consensus has been reached concerning what is cause and effect in the relation between hearing impairment and cognitive decline, several plausible mechanisms have been proposed that may explain the auditory-cognitive links − originally in the seminal work of Lindenberger and Baltes in the 1990s [18, 19]. The cognitive load theory states that speech perception in the presence of hearing impairment requires an increased allocation of cognitive resources, which over time may promote cognitive degeneration. Another possibility is that age-related hearing impairment and dementia have common causes, such as microvascular insufficiency or genetics. It has also been suggested that auditory deprivation, i.e., a reduction in output to the central auditory pathways, causes changes in the brain, which may affect cognitive abilities. In addition, auditory deprivation may indirectly increase the risk for dementia through social isolation and depression, referred to as the cascade theory [20]. Finally, many tasks in cognitive assessment contain verbal elements, thus relying on intact hearing [21]. Consequently, there is a risk for underestimating cognitive abilities among people with poor hearing. An up-to-date comprehensive taskforce report [22] on the sensory-cognitive relationship shows that the relationship is complex. So far, longitudinal as well as cross-sectional studies on the association of age-related hearing loss and cognitive function, impairment, and/or decline have been heterogeneous in terms of which methods were used to assess hearing function. For instance, numerous studies used pure-tone audiometry [8, 10, 12], which is regarded as an objective measure of peripheral hearing function, whereas other studies used self-report measures regarding perceived hearing problems [9, 14]. The latter may tap in to both peripheral and central hearing problems. In a study by Vassilaki et al. [13], the information about participants' hearing status was informant-based (reported by a spouse or equivalent), and in the study by Gurgel et al. [11], the presence of hearing loss was observed by the examiner conducting the cognitive assessment. Moreover, the use of speech-in-noise tests has been scarce in such studies. A further issue is that the cognitive tasks have also varied in the published literature, with some relying more heavily on auditory function and others on visual function. Few population-based studies have included and compared several measures of hearing function conducted on the same sample. Exploring the effect of a broader range of hearing measures on cognitive function in a relatively large, well-characterized population could serve to further our understanding of the nature of this relationship and inform the design of future studies.

### Aims of the Study

In the present study, we aimed to investigate the cross-sectional association between hearing and cognitive function in a representative sample of mainly community dwelling 70-year-old persons without dementia, using data from the most recent cycle of the Gothenburg H70 Birth Cohort Study 2014-2016. Additionally, we aimed to clarify whether different measures of hearing − pure-tone audiometry, speech-recognition-in-noise (SPRIN) audiometry, and self-reported hearing problems − yield different magnitudes of associations. We hypothesized that the speech-in-noise task and the self-report measure would show a stronger association with cognitive function than pure-tone audiometry would, given the fact that these involve central auditory function.

## Materials and Methods

### Study Population and Setting

The data used in the present study come from the Gothenburg H70 Birth Cohort Study, which is an ongoing prospective multidisciplinary study that examines the health and determinants of health in age-homogeneous representative samples of the older population in Gothenburg, Western Sweden. Gothenburg is the second largest city in Sweden with a population of 579,281 persons in 2019. During 2014-2016, a new birth cohort of 70-year-old persons born in 1944 was examined. All residents of Gothenburg that were aged 70 years at the time of invitation and born on dates ending in 0, 2, 5, or 8 were eligible for participation. A total of 1,203 persons responded (53.3% women, response rate 72.2%) and completed a detailed test battery covering a large range of health domains, including a hearing assessment (pure-tone audiometry and a questionnaire on perceived hearing problems), as well as a comprehensive cognitive evaluation. In addition, a systematically selected subsample (*n* = 251, response rate 82%) completed an extended audiological evaluation, which among other things involved a test of SPRIN. Further information about the methodologies of the main investigation [23, 24] and the substudy [25] is available in separate publications, including a more thorough description of the sample and the test protocol. The present analysis included all participants who participated in pure-tone audiometry and cognitive testing (total sample), and in speech-in-noise testing (subsample). Swedish language proficiency was not an explicit inclusion criterion, but participants were required to be able to communicate in Swedish in order to perform many of the tests. Exclusion criteria included having a confirmed dementia diagnosis according to DSM V criteria (*n* = 23). No further exclusion criteria were used in order to obtain a representative sample with a range of differing hearing and cognitive abilities. There were several different reasons for nonresponse. Importantly, 46 participants had not performed pure-tone audiometry, since this test was not included during domiciliary visits. Further reasons included being too tired, time constraints, or that the participant declined. The final sample consisted of 1,105 participants (52% women) and the subsample consisted of 247 participants (54% women). Procedure: the main investigation took place in the Neuropsychiatric Clinic at the Sahlgrenska University Hospital and lasted for a full day, including breakfast and lunch. Participants could also choose to perform the tests over 2 days. Specially trained research nurses administered most of the assessments. However, a mental health nurse, a psychiatrist, or a medical doctor conducted some of the cognitive tests. Moreover, the extended audiological examination (performed on a subsample) was conducted in a research facility within the Audiology Department at the University of Gothenburg by audiologists.

### Assessment of Cognitive Ability

The cognitive test battery was designed to cover a wide range of cognitive abilities and included tasks from the *Alzheimer's Disease Assessment Scale-Cognitive (ADAS-COG)* [26] as well as from the *Dureman and Sälde* [27] psychometric test battery, which were widely used in Sweden in the early 1970s when the H70 Study was first initiated. Instructions for the tests were given verbally and participants were encouraged to use hearing aids and spectacles as and when required. Most of the tests included a practice round to ensure that the participants had understood the instructions. Memory abilities were assessed with two tasks, one verbal and one nonverbal. The former involved delayed recall of 12 objects, and the latter involved delayed recall of five images. Working memory was measured with a supra-span memory test, where participants were asked to repeat a list of items of clothing. To test semantic fluency, participants had to orally produce as many animals as possible, and to test phonemic fluency, participants had to generate as many words beginning with /F/, /A/, and /S/ as possible, in 60 s. Mental speed was measured through a figure identification task, where participants needed to identify which image out of five was repeated. To assess logical reasoning, participants were asked to identify which figure out of five differed from the rest. Finally, visuospatial abilities were tested through a building block task, in which participants were required to produce a block design that matched that of a given model. More detailed information about these tests, including their validity and scoring procedures, is available elsewhere [23]. Raw test scores were z-transformed and global cognitive function was defined as the average of these, given that valid scores were available in at least four domains. The internal consistency of the global index was acceptable (Cronbach's alpha = 0.72).

### Assessment of Hearing

#### Self-Reported Hearing

Self-reported hearing was examined with a 10-item questionnaire that has been included in the H70 Study since its beginning. The questionnaire was given to all participants of the study during a general health interview conducted by one of the nurses. The questions were designed to measure hearing-related problems in a number of situations. For the purpose of the present study, a *hearing index* based on five items was selected to represent self-reported hearing problems. This index has been described and validated against pure-tone audiometry in a previous study [28]. The first item concerned overall hearing ability and was phrased: “*How is your hearing?*”. Three response categories were possible: “Fine, no problems,” “Slightly impaired,” or “Significantly impaired.” The remaining four questions concerned perceived hearing problems in the following situations: conversations with one other person; conversations in a group; listening to the television; and hearing the doorbell. Three response categories were available for all four items: “No problems,” “Slight problems,” and “Significant problems.” A score of 0, 1, or 2 points were given for each item, yielding a possible score of 0–10, where 10 indicated significant problems in all situations, etc. In addition, participants were also asked about the presence of tinnitus (of any degree) and hearing aid use.

#### Pure-Tone Audiometry

Pure-tone air conduction thresholds were measured in a quiet office with an Entomed SA 202IV audiometer and Sennheiser HDA200 circumaural headphones, using an automated computerized test procedure. Ambient noise levels were measured and found to be within the tolerance values specified in ISO 8253-1:2010, Tables 2 and 3 relevant standards [29]. Additionally, subjective controls were performed regularly, as described in clause 11.2 of the same standard. The test always started in the right ear at 1 kHz with a familiarization procedure, to ensure compliance with the method. If acceptable, the threshold-seeking procedure then began using a bracketing technique. Thresholds were defined as the lowest level at which the test tone was reliably detected 3 times, and were determined for eight discrete test frequencies (0.25–8 kHz) in the measurement range of 0–90 dB HL. The average of pure-tone thresholds across 0.5, 1, 2, and 4 kHz was calculated (PTA4) for both ears, and the ear with the lowest (in dB HL) PTA4 was considered the better ear. Hearing loss was categorized as none (0–25 dB HL), mild (26–40 dB HL), moderate (41–60 dB HL), or severe (>60 dB HL). The severe category was amalgamated with the moderate category, since it only contained two participants.

#### Speech-in-Noise Audiometry

A test of SPRIN [30] was completed by the subsample that participated in the extended audiological investigation. The test is a clinical standard measurement in Sweden that mainly tests peripheral hearing function. An Equinox audiometer with Telephonics TDH-39 headphones was used, and the test was performed in a soundproofed booth complying with international standards [29] in terms of calibration and maximum permissible ambient noise levels. Phonemically balanced lists of 50 monosyllabic words − read by a male speaker − were presented monaurally, starting with the better ear. The initial presentation level was set to 35 dB above the estimated speech threshold (based on the three-frequency pure-tone average of 0.5–2 kHz) but was individually adjusted to achieve adequate comfort and audibility. An unmodulated speech-weighted noise was presented simultaneously at a fixed signal-to-noise ratio of +4 dB. The results were expressed as the percentage of correctly identified words, henceforth referred to as the SPRIN score. If participants could not identify any of the first 10 words, a score of zero was given.

#### Confounding Variables

As part of the main investigation, data regarding various health parameters were collected. Systolic and diastolic blood pressure was recorded in the right arm with the participant in sitting position, after 5 min of rest. Hypertension was defined as a systolic blood pressure ≥140 mm Hg or diastolic blood pressure ≥90 mm Hg, and/or current treatment with antihypertensive drugs. Cardiovascular disease (past or present) was diagnosed if any of the following were present: angina pectoris (self-reported or presence of chest pain according to the ROSE questionnaire), myocardial infarction (self-reported or presence of major or moderate Q-waves on the ECG), or heart failure (self-reported). For more information regarding these health variables, see Rydberg Sterner et al. [23].

### Statistical Analysis

Statistical analysis was performed using the R studio version 1.2 and SPSS for Windows version 25. Comparisons between the total sample and the subsample, and between responders and nonresponders were performed using χ^2^ tests. Nonresponders refer to participants in the birth cohort that were not selected for the study samples (due to not having available cognitive and/or hearing data). Multiple linear regression was used to study the associations between global and domain-specific cognitive function and three different measures of hearing. Since the objective was to compare the extent to which each parameter explained the variance in the outcome variable, the standardized regression coefficients (β) were used. The 95% confidence interval (CI) of the β coefficients were calculated and used to evaluate statistically significant differences between regression coefficients. The main dependent variable, global cognitive function, was examined with a histogram and a normal Q-Q plot and was found to be normally distributed, both in the main sample and in the subsample. Three models were created, in which the following explanatory variables were used: model 1 − the pure-tone average (PTA4) in the better hearing ear; model 2 − the SPRIN score in the better hearing ear, defined as the ear with the lowest (in dB HL) PTA4; model 3 − self-reported hearing index on a continuous scale. All three models were adjusted for years of education (continuous variable) and sex, hypertension, cardiovascular disease, and tinnitus (all binary variables). In secondary analyses, we adjusted model 2 for PTA4 and created a model with all three hearing measures added simultaneously. Additionally, we performed the regression analyses for men and women separately. The significance of each model was tested with ANOVA and the assumptions of homoscedasticity and independence were ascertained by plotting standardized residuals against predicted scores, and by normality analysis of the residuals. Based on the Collinearity Diagnostics in SPSS, collinearity between the independent variables was only moderate (VIF values <2) and was therefore not assumed to affect the results. The significance level for all analyses was set at *p* < 0.05 and missing data were excluded listwise.

## Results

### Demographic, Hearing, and Cognitive Characteristics of the Sample

Demographic and general health characteristics of the sample are presented in Table 1. The sample consisted of 1,105 persons aged 70 years (52% women) without dementia, with a mean educational attainment of 13 years (SD = 4 years). Twenty percent of the sample had only completed basic education (≤9 years), whereas more than half had higher-level education (>12 years). According to the definitions used in the present study, hypertension and cardiovascular disease affected 71% and 13% of participants, respectively. The distribution of these variables in the subsample that performed speech-in-noise testing (*n* = 247) did not differ significantly from the sample, except concerning educational attainment, where the subsample were slightly more highly educated (mean difference = 0.6 years, *p* = 0.031). Additionally, the participants of the present study did not differ significantly from the remainder of the birth cohort, with regard to the abovementioned characteristics.

**Fig. 1:**
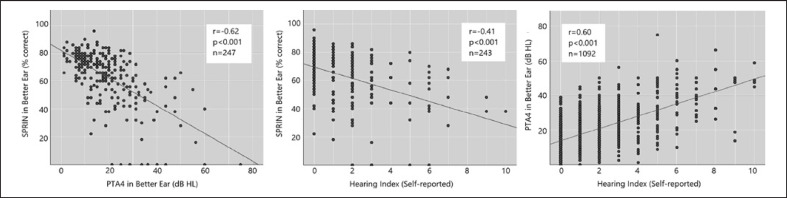
Correlation between the three different hearing measures used in the present study: PTA4 in the better ear, SPRIN score in the better ear, and a self-reported hearing index.

Detailed descriptive characteristics of all hearing and cognitive variables are available in the online supplementary Material (for all online suppl. material, see www.karger.com/doi/10.1159/000527930). Global cognitive scores were available in 99% of the participants and were normally distributed in the sample, with even distribution among different hearing loss categories (histograms available in Fig. S1). Moreover, 4% of female and 9% of male participants scored <−1 SD from the mean, and 0.3% (female) and 1.7% (male) scored < −1.5 SD from the mean, which are commonly used criteria for possible mild cognitive impairment. Hearing loss in the better hearing ear was present in 25% of the sample, of which 20% had mild degree (PTA4 = 21–40 dB HL) and 5% had moderate degree (PTA4 = 41–60 dB HL). Only two participants had severe degree (>60 dB HL). Furthermore, 31% of the sample reported having tinnitus, and 13% reported using hearing aids (online suppl. Table. S1. The interrelations between subjectively measured hearing (hearing index) and the two objective measures of hearing (PTA4 and SPRIN) are illustrated in Figure 1. A strong negative correlation was found between the PTA4 in the better ear and the SPRIN score in the better ear (*r* = −0.62, *p* < 0.001). A strong correlation was also found between the self-reported hearing index and the PTA4 in the better hearing ear (*r* = 0.60, *p* < 0.001), whereas a moderate correlation was found between the hearing index and the SPRIN score (*r* = −0.41, *p* < 0.001).

### Association of Hearing with Global Cognitive Function

**Fig. 2:**
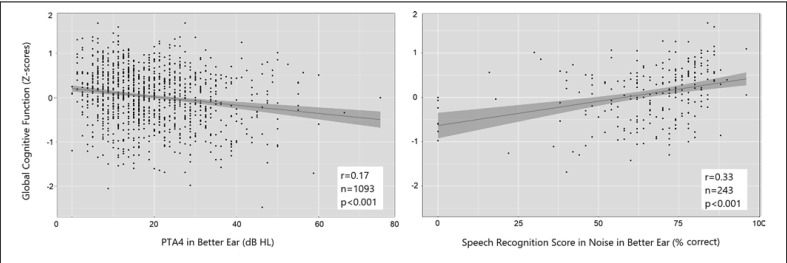
Scatter plots illustrating the association of two measures of hearing: pure-tone audiometry (left plot) and SPRIN (right plot) with global cognitive function in a population-based sample of community dwelling 70-year-olds free from dementia.

Significant associations were found between the objective hearing measures − PTA4 (β = −0.13, 95% CI, −0.18, −0.07) and SPRIN (β = 0.30, 95% CI, 0.19, 0.40) − and global cognitive function, adjusting for sex, educational attainment, hypertension, cardiovascular disease, and tinnitus (see Table 3 and Fig. 2 for details). No association with global cognitive function was found for self-reported hearing loss (β = −0.02, 95% CI, −0.07, 0.03). The strength of the association with global cognitive function was significantly higher for SPRIN than for the PTA4, based on their CIs not overlapping. Although significant, none of the models explained the variance in global cognitive scores to any large extent, 18.9% for PTA4 and 22% for SPRIN (adjusted R^2^). In additional analyses, model 2 (using SPRIN as the main explanatory variable) was adjusted for PTA4, but this did not alter the regression coefficient for SPRIN. When adding all three hearing measures simultaneously in a model (not in any table), only SPRIN remained a significant predictor (β = 0.32, *p* < 0.001). Due to possible issues with collinearity, these models are not presented in any table.

#### Hearing Aid Use and Gender

**Fig. 3:**
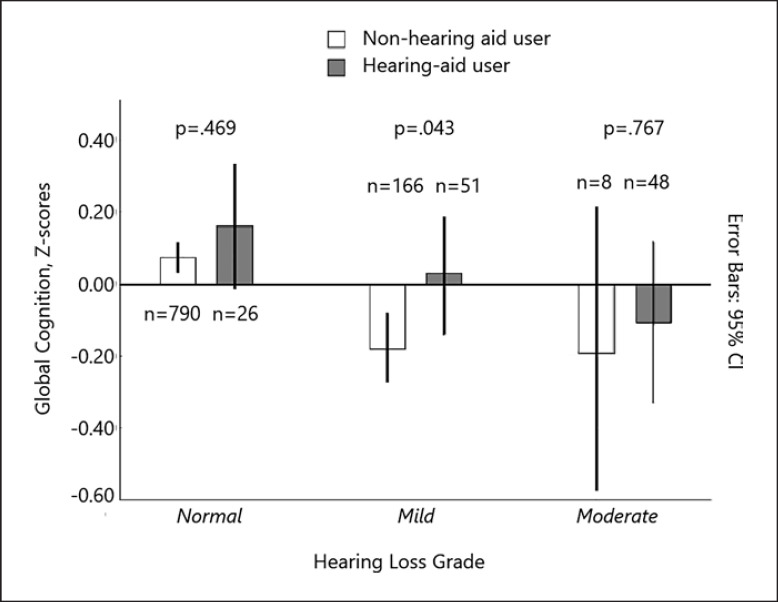
Mean global cognitive function in participants with and without hearing aid, split by hearing loss categories (PTA4 in the better ear, normal: 0–25 dB HL; mild: 26–40 dB HL; moderate >40 dB HL; NB. Only two participants had severe loss and were thus included in the moderate category).

When analyzing mean global cognitive scores in different hearing loss categories (Fig. 3), hearing aid users showed better cognitive function overall than nonusers, but the difference was only significant in the category of mild hearing loss (mean difference of 0.2 points, *p* = 0.043). Reporting tinnitus was not associated with global cognitive function in the bivariate analysis (crude β = 0.05, 95% CI, −0.01, 0.11) and only weakly in models that included PTA4 and self-reported hearing loss. Being female was associated with higher global cognitive function (crude β = 0.14, 95% CI, 0.08, 0.20), an effect which remained in the models with PTA4 and self-reported hearing loss as main explanatory variables, but interestingly not in the model that included SPRIN (Table 2). We also performed the regression analyses for men and women separately, but no significant differences were found in the β coefficients (not in any table).

### Association of Hearing with Domain-Specific Cognitive Function

Both objective hearing measures (PTA4 and SPRIN) were significantly associated with worse scores in all cognitive domains (adjusted for sex, education, cardiovascular factors, and tinnitus), except for visual memory in the case of PTA4, and visual and working memory in the case of SPRIN (Table 3). The strengths of the associations were in the range of β = −0.11 to β = −0.07 for PTA4 and β = 0.12 to β = 0.22 for SPRIN. Self-reported hearing ability was only associated only with working memory and mental speed (range: β = −0.06 to β = −0.05). Additionally, verbally loaded tests yielded slightly stronger associations with SPRIN, than did nonverbally loaded tests. No such pattern was seen for PTA4. Detailed regression results are available in Table 3.

## Discussion

### Main Findings

In this population-based sample of 70-year-old individuals free from dementia, poorer hearing function was independently associated with worse global and domain-specific cognitive function, in line with previous research [10, 31]. Furthermore, we demonstrated that the association of hearing with cognitive function only applied when analyzing objective measures of hearing, i.e., pure-tone audiometry and SPRIN scores. The self-reported hearing measure did not yield any significant association with global cognition, and only weak associations with some of the cognitive domains (working memory, visual memory). These findings bear relevance to the design of future population-based studies on the relation between hearing and cognition, since hearing function is commonly estimated with self-report measures in such studies. Moreover, we demonstrated that the SPRIN score was better at predicting cognitive function than the pure-tone average was, which was an expected finding given the well-established role of cognition in speech processing [6]. This may suggest that speech-in-noise tests could be considered as a means of assessing hearing function in epidemiological studies. Such tests are performed at suprathreshold levels and are therefore less sensitive to background noise in the test environment. Additionally, some speech-in-noise tests, such as adaptive ones, may also be easier to carry out by nonaudiologist personnel. However, a bidirectional association probably exists between cognitive function and speech-in-noise recognition, which complicates the interpretation. Moreover, several different speech-in-noise tests exist, using, e.g. digits, syllables, words, or sentences as stimuli. Consequently, the linguistic and cognitive loads of the tests vary, as well as the extent to which they rely on central auditory processing skills, which would need to be considered. Finally, the fact that both verbally loaded and nonverbally loaded cognitive tasks were independently associated with the objective hearing measures, albeit weakly, suggests a broad relationship between hearing and cognition. Taken together, the results of the present study add to the current knowledge base by offering a more in-depth analysis than what has been included in most of previous population-based studies, of how different hearing measures and different cognitive measures interrelate with one another.

### Comparison with Previous Research

Contrary to our findings, self-reported hearing loss − assessed with a variety of different questions (usually dichotomized) − has been shown to correlate with cognitive function in previous research [14] and has been associated with accelerated decline in cognitive function in longitudinal studies [9]. Generally, self-reported measures of hearing loss are known to correlate reasonably well with pure-tone audiometry [28], which was also corroborated in the present study. However, it has also been demonstrated that older persons tend to overestimate their hearing ability and, conversely, that younger persons underestimate theirs [32], which further supports that psychoacoustic measures should be used when studying age-related hearing in epidemiological studies, rather than self-report measures. On a separate note, the objective hearing measures used in the present study − pure-tone audiometry and SPRIN − are predominantly tests of peripheral auditory function. Several studies have shown a stronger effect on cognitive function when central auditory function is considered, e.g., reference [33]. Bearing in mind that older persons often experience significant problems with speech comprehension, sometimes even when peripheral hearing sensitivity is preserved (presumably caused by age-related decline in the central auditory pathways), we expected that the self-report measure would correlate more strongly with cognitive function than pure-tone audiometry would, contrary to our actual findings. This may be explained by the age composition of the present sample (all participants are 70 years), since central auditory dysfunction is more widespread in the “older old.” There is ample evidence from experimental research that poor performance on speech-in-noise tests cannot be explained by the presence of hearing loss alone [6, 33]. In support of this notion, we found in our study that the association of the SPRIN score with global cognitive function persisted even after adjusting for PTA4 (not presented in any table). In fact, PTA4 was not a significant predictor of cognitive function in the presence of the SPRIN score. It is also important to note that complex associations may exist between the different measures of hearing, something that was not accounted for in the present study. Along this line, it was demonstrated in a recent publication by Hämäläinen et al. [34] that self-reported sensory ability is associated with both sensory and nonsensory factors (e.g., age, gender, and comorbidities).

Moreover, in a meta-analysis [35] of experimental studies on the relation between speech-in-noise measures and cognitive function, an overall pooled association of ∼0.3 was reported. Interestingly, this figure agrees well with what we found in our study (β = 0.34.). The strength of the association of the SPRIN score with global cognitive function in our study was more or less equal to that of educational attainment, which is a well-known risk factor of cognitive impairment. In fact, in the model with the SPRIN score as the main explanatory variable (*model 2* in Table 2), only educational attainment remained a significant predictor of global cognitive function. Jointly, these two variables explained almost as much variance in cognitive scores as did the full model that included the other confounders. Furthermore, the results of the present study agree well with the findings of other population-based studies that have analyzed speech-in-noise performance in relation to domain-specific and global cognitive function [16, 36].

### Cognitive Function in Users and Nonusers of Hearing Aids

Whether or not hearing aid use can mitigate the effect of hearing loss on cognitive performance, and progression of cognitive decline, is an essential question that has been addressed in numerous studies with mixed results [9, 19, 37]. The findings from the present study cannot answer this question due to the cross-sectional design, but there was at least some indication of better cognition in hearing aid users (evident from Fig. 3). Notably, hearing aids were only used by 10% of participants in the present sample, whereas hearing loss (bilateral) was measured in 25%. In future studies, it would be of great interest to include data on number of hours of usage and whether hearing aids were fitted bilaterally or not. The effect of tinnitus on cognitive function has also been investigated in a number of studies, with results suggesting that tinnitus is related to worse performance in some cognitive domains, such as attention [38]. The present study was not designed to evaluate the effect of tinnitus on cognitive function, but the indication was that the presence of tinnitus was not associated with cognitive function (Table 2). However, the frequency and degree of tinnitus would need to be considered to draw any conclusions, as would the impact of other related factors, such as anxiety, depression, and tinnitus degree and duration.

### Strengths and Limitations

The design of the Gothenburg H70 Birth Cohort Study offers unique opportunities to study multiple aspects of health and its determinants in age-homogeneous samples of older people, allowing for comprehensive descriptions of the relationship between various age-related health entities at specific stages in the aging process. Using age-homogeneous samples has been shown to be an advantage in cross-sectional analyses, since age-related variables may appear more correlated than they truly are when samples contain mixed ages [39]. Here, we were able to explore the association between hearing and cognition in early old age specifically, which is valuable as a reference for future longitudinal studies. A key strength of the present study is that both hearing and cognitive function were measured thoroughly with a battery of standardized tests. In many previous studies, cognitive status has been estimated with screening instruments, such as the Mini-Mental State Examination or the Montreal Cognitive Assessment [8, 9, 33]. While these may be sensitive to dementia, they are not designed to distinguish between varying degrees of cognitive ability and may yield ceiling effects. Correspondingly, the hearing assessments used in previous similar studies have often been basic. The fact that we included both subjective and objective hearing measures in the present study, and that we used gold-standard tests that were conducted in accordance with clinical standards, is therefore an advantage that adds significantly to the quality of the study. Naturally, there were also some limitations of the study. First, there were remarkably few participants with severe hearing loss (*n* = 2, 0.18%) and none with profound hearing loss in the sample, which may be an effect of improved hearing status in this birth cohort in comparison to previous generations [24]. Second, the fact that SPRIN was only available in a subsample (constituting approximately 20% of the total sample) may have decreased the opportunity to compare between the various hearing measures. Furthermore, it is possible that better and more comprehensive self-report measures exist than the one used here, for instance, the widely used Hearing Handicap Inventory for the Elderly (HHIE) [40]. However, since one of the goals of the Gothenburg H70 Birth Cohort Studies is to allow for the study of cohort effects, the reporting measures in the study have not been changed much since the beginning of the study in 1971, to allow for accurate comparisons.

Moreover, in the endeavor of achieving representativeness, the sample and subsample studied in the present study included persons who did not have Swedish as their first language. There is a possibility that this may have affected the results, both in terms of speech-in-noise performance and in the more verbally loaded cognitive tasks. Additionally, considering the effect of the visual status of the participants could have enhanced the conclusions on how sensory and cognitive functions interact. Finally, the present analysis was cross-sectional and it is not possible to assess the direction of the relationship between hearing and cognition. Although there is evidence to support that hearing loss precedes cognitive decline, it is certainly possible that poor cognitive abilities lead to worse hearing outcomes as well.

### Implications

In all, these results highlight the importance of considering hearing loss as a risk factor of cognitive impairment, as recognized in the Lancet report by Livingston et al. [17]. In spite of a recent surge in the research output on the role of hearing loss in the risk of cognitive impairment, hearing is frequently overlooked in dementia research and in geriatric healthcare. Furthermore, not appropriately controlling for hearing ability in studies investigating the risk of cognitive impairment may produce misleading results. For instance, female gender was associated with better global cognitive function in the present study, until adjusting for the SPRIN score, suggesting that the presumed effect of gender was explained by the variance in speech recognition ability. Moreover, the indications of better cognitive function in hearing aid users seen in the present study may justify efforts for earlier identification of age-related hearing loss and recommendations of hearing aids even in cases of mild hearing loss. Hearing aids are a cost-effective intervention, with benefits that extend beyond improved communication, including amelioration of tinnitus and possibly improved cognitive health [9].

## Conclusions

Objectively assessed hearing function, using pure-tone audiometry and SPRIN testing, is independently associated with global and domain-specific cognitive function in early old age. Self-reported hearing loss, on the other hand, may be too insensitive a measure to study the relationship. Hearing aid users had better cognitive scores among persons with mild hearing loss. However, more research is needed to study the role of hearing aid use in relation to age-related cognitive declines.

## Statement of Ethics

Written informed consent was obtained from all participants or their relatives. The Regional Ethical Review Board in Gothenburg reviewed and approved the study protocols of the main investigation (reg. no. 869-13) and the substudy (reg. no. 976-13).

## Conflict of Interest Statement

The authors have no conflicts of interest to declare.

## Funding Sources

This study was supported by grants from Rune och Ulla Amlövs Stiftelse (2016-105), Felix Neuberghs Stiftelse (2020-0218), Foundation Agneta Prytz-Folke and Gösta Folke (2013-0613), Region Västra Götaland (2014-0601), The Swedish Research Council (No. 2012-5041, 2013-8717, 2015-02830), Swedish Research Council for Health, Working Life and Welfare (2013-1202, AGECAP 2013-2300, 2013-2496, 2013-0475, 2018-00471), the Swedish state under the agreement between the Swedish government and the county councils, the ALF-agreement (ALF 716681), Konung Gustaf V:s och Drottning Victorias Frimurarestiftelse, Swedish Alzheimer Foundation, Hjärnfonden, Eivind och Elsa K:son Sylvans Stiftelse, Stiftelsen Söderström-Königska Sjukhemmet, Stiftelsen för Gamla Tjänarinnor, Handlanden Hjalmar Svenssons Forskningsfond, and Stiftelsen Professor Bror Gadelius Minnesfond. None of the funding sources had any role or input into the design and conduct of the study or approval of the manuscript.

## Author Contributions

Maria Hoff and Johan Skoog conceived the design and analyzed the data. Maria Hoff performed the literature review and wrote the first draft of the article. Maria Hoff, Tomas Tengstrand, Johan Skoog, and Timothy Hadarsson Bodin collected the data. Ulf Rosenhall, Ingmar Skoog, and André Sadeghi contributed to the design, analysis, and interpretation of findings, and supervised the research project. All authors revised the first draft and approved the final version of the manuscript.

## Data Availability Statement

All data generated or analyzed during this study are included in this article and/or its supplementary material files. Further inquiries can be directed to the corresponding author (M.H.).

## Supplementary Material

Supplementary data

Supplementary data

## Figures and Tables

**Table 1:** Distribution of selected demographic and health variables in the study sample (*n* = 1,105), as well as its statistical difference from the subsample (*n* = 247) that performed a SPRIN test, and nonresponders (*n* = 98)

	*N* (%)	Difference from	
		Subsamplexyx*	Non-resp.**
Sex			
Male	517 (47)	*p* = 0.421	*p* = 0.271
Female	588 (53)		
*Missing*	*None*		
Educational attainment			
≤9 years	225 (20)	*p* = 0.046	*p* = 0.233
10–12 years	319 (29)		
>12 years	552 (50)		
*Missing*	*9 (1)*		
Hypertension			
Yes	780 (71)	*p* = 0.813	*p* = 0.736
No	324 (29)		
*Missing*	*1(0)*		
Cardiovascular disease			
Yes	147 (13)	*p* = 0.145	*p* = 0.432
No	954 (86)		
*Missing*	*4 (1)*		
Degree of hearing loss^a^			
Normal	827 (75)	*p* = 0.535	N/A
Mild	221 (20)		
Moderate	55 (5)		
Severe	2 (0)		
*Missing*	*None*		
Tinnitus			
Yes	340 (31)	*p* = 0.118	*p* = 0.529
No	765 (69)		
*Missing*	*none*		
Hearing aid use			
Yes	127 (11)	*p* = 0.578	*p* = 0.444
No	971 (88)		
*Missing*	7 *(1)*		

Non-resp., nonresponders; N/A, not applicable.

* Comparison between subsample (*n* = 247) and the remainder of the total sample (*n* = 858), using a χ^2^ test.

** Comparison of the sample with nonresponders, i.e., the remainder of the birth cohort, using a χ^2^ test. No pure-tone audiometry results were available in nonresponders, which is why N/A shows under *degree of hearing loss.*

a Degree of hearing loss is based on the average of pure-tone thresholds across 0.5–4 kHz, in the better hearing ear, normal: 0–25 dB HL; mild: 26–40 dB HL; moderate: 41–60 dB HL; severe: >60 dB HL.

**Table 2:** Multiple regression results for global cognitive function on three different measures of hearing function: the pure-tone average of 0.5–4 kHz in the better hearing ear (model 1), the SPRIN score in the better hearing ear (model 2), and self-reported hearing problems (model 3)

	Crude β (95% CI)	*p* value	Model 1		Model 2		Model 3	
			Adj. β (95% CI)	*p* value	Adj. β (95% CI)	*p* value	Adj. β (95% CI)	*p* value
Explanatory variables								
PTA4, better ear	−0.17 (−0.23, −0.11)	<0.001	−0.13 (−0.18, −0.07)	<0.001	−	−	−	−
SPRIN, better ear	0.33 (0.21, 0.45)	<0.001	−	−	0.30 (0.19, 0.40)	<0.001	−	−
Self-rated hearing	−0.01 (−0.06, 0.05)	0.405	−	−	−	−	−0.02 (−0.07, 0.03)	0.466
Adjusting variables								
Years of education	0.38 (0.33, 0.44)	<0.001	0.37 (0.32, 0.42)	<0.001	0.34 (0.22, 0.46)	<0.001	0.39 (0.34, 0.45)	<0.001
Being female	0.14 (0.08, 0.20)	<0.001	0.15 (0.09, 0.20)	<0.001	0.07 (−0.05, 0.19)	0.238	0.14 (0.09, 0.19)	<0.001
Being hypertensive	−0.07 (−0.13, −0.01)	0.024	−0.02 (−0.07, 0.03)	0.456	0.09 (−0.03, 0.20)	0.138	−0.03 (−0.08, 0.03)	0.305
Having CVD	−0.08 (−0.14, −0.02)	0.008	−0.05 (−0.10, 0.00)	0.073	0.00 (−0.10, 0.10)	0.984	−0.05 (−0.10, 0.00)	0.070
Having tinnitus	0.05 (−0.01, 0.11)	0.080	0.08 (0.02, 0.13)	0.005	0.08 (−0.04, 0.19)	0.213	0.06 (0.01, 0.12)	0.026
		*Adjusted R2*	*0.189*		*0.217*		*0.179*	
		*F*	*43*		*12*		*41*	
		*Significance of F*	*p < 0.001*		*p* < 0*.001*		*p* < 0*.001*	
		*Valid N*	1,076		*235*		1,076	

Dependent variable: global cognitive function (average of z-transformed raw scores from a cognitive test battery). Each model was adjusted for years of education, sex, hypertension, cardiovascular disease, and tinnitus. Adj., adjusted; PTA4, pure-tone average of 0.5–4 kHz (dB HL); SPRIN, speech-recognition-in-noise (% correct); CVD: cardiovascular disease.

**Table 3:** Multivariate multiple regression analyses of the association between domain-specific cognitive function and hearing, measured with pure-tone audiometry (*n* = 1,105), a test of SPRIN (*n* = 247), and a self-report measure (*n* = 1,105)

*Dependent variables*	Independent variables					
	Better ear PTA4		Better ear SPRIN score		Self-reported hearing ability	
Cognitive domain (*Task*)	Adj. β* (95% CI)	*p* value	Adj. β* (95% CI)	*p* value	Adj. β* (95% CI)	*p* value
Verbal tests						
Semantic fluency (*listing animals*)	−0.08 (−0.14, −0.02)	0.010	0.20 (0.10, 0.38)	0.002	0.02 (−0.07, 0.08)	0.515
Phonemic fluency (*listing objects*)	−0.08 (−0.14, −0.02)	0.010	0.22 (0.09, 0.37)	0.001	−0.05 (−0.10, 0.01)	0.081
Episodic memory (*delayed word recall*)	−0.11 (−0.18, −0.04)	<0.001	0.21 (0.08, 0.37)	0.002	−0.03 (−0.08, 0.04)	0.238
Working memory (*supraspan memory*)	−0.07 (−0.14, 0.00)	0.017	0.12 (−0.01, 0.24)	0.081	−0.05 (0.00, 0.11)	0.045
Nonverbal tests						
Inductive reasoning (*figure logic*)	−0.08 (−0.16, −0.00)	0.013	0.14 (0.02, 0.32)	0.041	−0.03 (−0.10, 0.01)	0.331
Visuospatial ability (*block design*)	−0.08 (−0.15, −0.01)	0.005	0.18 (0.00, 0.32)	0.010	0.03 (−0.08, 0.04)	0.345
Mental speed (*figure identification*)	−0.08 (−0.15, −0.00)	0.007	0.19 (0.01, 0.30)	0.007	−0.06 (−0.10, 0.01)	0.037
Episodic memory (*picture memory*)	−0.04 (−0.11, 0.01)	0.170	0.09 (−0.01, 0.29)	0.198	0.04 (−0.01, 0.11)	0.060

PTA4, pure-tone average of 0.5–4 kHz (dB HL); SPRIN, speech-recognition-in-noise; Adj., adjusted.

* Adjusted for sex, education, cardiovascular disease, hypertension, and tinnitus.
