# Supplementary material for: Hearing Loss and Cognitive Function in Early Old Age: Comparing Subjective and Objective Hearing Measures
Source: Gerontology. 2022 Dec 14;69(6):694–705. doi: 10.1159/000527930 (PMC10273901; doi:10.1159/000527930)
Supplement: Supplementary file 1 — Supplementary data [file ger-0069-0694-s01.docx]

**Table S1**: Descriptive characteristics of hearing and cognitive variables in the whole sample, and split by degree of hearing loss, based on the pure-tone average in the better hearing ear.

|  |  |  |  |  | Grade of Hearing Loss: | | | | | | | | | | |
| --- | --- | --- | --- | --- | --- | --- | --- | --- | --- | --- | --- | --- | --- | --- | --- |
|  | Total | | |  | Normal, 0-25 dB HL | | |  | Mild, 26-40 dB HL | | |  | Moderate/Severe, >40 dB HL | | |
| *Hearing variables* | M (SD) | Min | Max |  | M (SD) | Min | Max |  | M (SD) | Min | Max |  | M (SD) | Min | Max |
| PTA4 Better Ear (dB HL) | 19.0 (11.4) | 0 | 75 |  | 13.7 (6.3) | 0 | 25 |  | 31.2 (10.2) | 26 | 40 |  | 48.5 (6.5) | 41 | 75 |
| PTA4 Worse Ear (dB HL) | 25.6 (14.4) | 3 | 88 |  | 19.9 (10.1) | 3 | 85 |  | 38.6 (4.0) | 26 | 84 |  | 56.1 (9.5) | 41 | 88 |
| SPRIN Better Ear (%) | 63.0 (19.1) | 0 | 96 |  | 68.4 (14.3) | 0 | 96 |  | 48.0 (18.8) | 0 | 82 |  | 36.6 (24.1) | 0 | 66 |
| SPRIN Worse Ear (%) | 60.0 (20.1) | 0 | 96 |  | 65.3 (16.3) | 0 | 96 |  | 47.6 (20.2) | 0 | 82 |  | 27.7 (21.6) | 0 | 54 |
| Self-rated Hearing Index | 1.5 (2.0) | 0 | 10 |  | 0.9 (1.3) | 0 | 9 |  | 2.6 (2.1) | 0 | 8 |  | 5.6 (2.4) | 1 | 10 |
|  |  |  |  |  |  |  |  |  |  |  |  |  |  |  |  |
| Overall hearing problems* | Percent | (N) | |  | Percent | (N) | |  | Percent | (N) | |  | Percent | (N) | |
| *Fine, no problems* | 54.4 % | 598 | |  | 66.7 % | 550 | |  | 22.0 % | 48 | |  | 0.0 % | 0 | |
| *Mild problems* | 36.9 % | 405 | |  | 31.4 % | 259 | |  | 59.6 % | 130 | |  | 28.1 % | 16 | |
| *Significant problems* | 8.7 % | 96 | |  | 1.8 % | 15 | |  | 18.3 % | 40 | |  | 71.9% | 41 | |
|  |  |  | |  |  |  | |  |  |  | |  |  |  | |
| *Cognitive variables* | M (SD) | Min | Max |  | M (SD) | Min | Max |  | M (SD) | Min | Max |  | M (SD) | Min | Max |
| Global Function, Z-Score | 0.0 (0.6) | -2.5 | 1.7 |  | 0.1 (0.6) | -2.1 | 1.7 |  | -0.1 (0.6) | -2.0 | 1.7 |  | -0.1 (0.7) | -2.5 | 1.2 |
| Semantic Fluency | 24.0 (6.4) | 0.0 | 49.0 |  | 24.3 (6.2) | 6 | 49 |  | 22.9 (6.5) | 6 | 41 |  | 23.7 (6.3) | 8 | 47 |
| Phonemic Fluency | 40.8 (14.6) | 0.0 | 110.0 |  | 41.7 (14.5) | 4 | 110 |  | 38.1 (14.1) | 2 | 78 |  | 39.4 (15.8) | 3 | 84 |
| Memory (Verbal) | 7.9 (1.7) | 0.0 | 12.0 |  | 8.0 (1.7) | 2 | 12 |  | 7.6 (1.8) | 0 | 12 |  | 7.5 (1.8) | 2 | 12 |
| Working Memory | 7.6 (1.5) | 2.0 | 10.0 |  | 7.7 (1.5) | 2 | 10 |  | 7.5 (1.6) | 2 | 10 |  | 7.1 (1.6) | 3 | 10 |
| Logical Reasoning | 19.8 (4.4) | 0.0 | 30.0 |  | 20.0 (4.2) | 0 | 29 |  | 19.0 (4.4) | 3 | 30 |  | 19.0 (4.9) | 2 | 27 |
| Visuospatial ability | 20.5 (7.0) | 0.0 | 41.0 |  | 21.0 (6.7) | 0 | 39 |  | 19.2 (7.5) | 0 | 41 |  | 20.1 (8.2) | 0 | 36 |
| Mental Speed | 27.3 (8.0) | -2.5 | 57.5 |  | 27.7 (7.8) | 3.5 | 57.5 |  | 26.2 (8.2) | 4.5 | 53.8 |  | 26.3 (8.0) | -2.5 | 42.5 |
| Memory (Visual) | 22.0 (4.1) | 2.0 | 28.0 |  | 22.1 (4.0) | 6 | 28 |  | 21.6 (4.5) | 2 | 28 |  | 21.4 (3.7) | 13.0 | 28.0 |
|  |  |  |  |  |  |  |  |  |  |  |  |  |  |  |  |

*Based on item 1 in the hearing questionnaire*: ‘How is your hearing?’*

*PTA4: Pure-tone average of 0.5-4 kHz; dB HL: Decibel hearing level; SPRIN: Speech Recognition in Noise*
