# Supplementary figures and images for: Hearing Loss and Cognitive Function in Early Old Age: Comparing Subjective and Objective Hearing Measures
Source: Gerontology. 2022 Dec 14;69(6):694–705. doi: 10.1159/000527930 (PMC10273901; doi:10.1159/000527930)

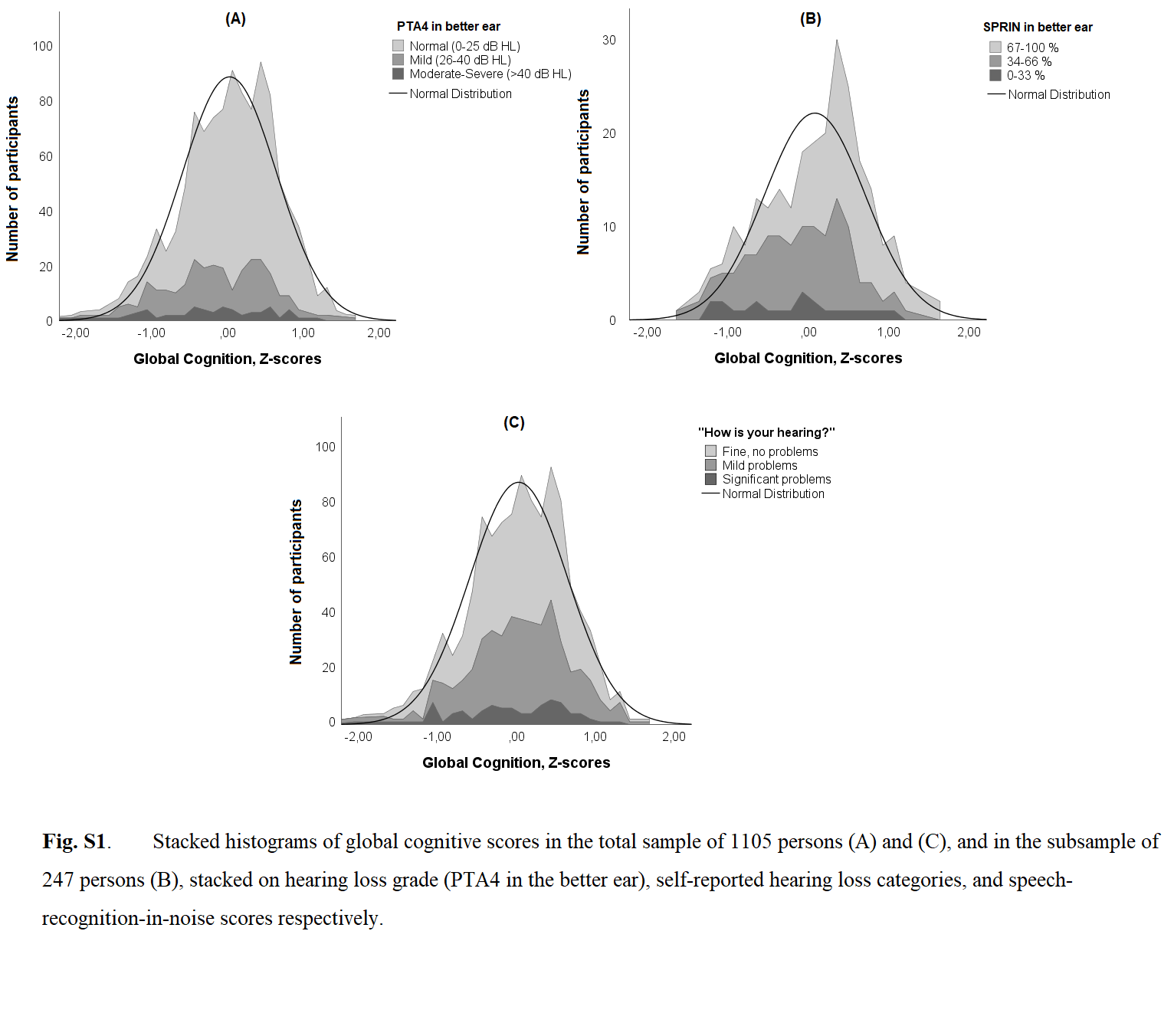

Supplement: Supplementary file 2 — Supplementary data [file ger-0069-0694-s02.png]
